# Quality of care in the intensive care unit from the perspective of patient’s relatives: development and psychometric evaluation of the consumer quality index ‘R-ICU’

**DOI:** 10.1186/s12913-016-1975-4

**Published:** 2017-01-24

**Authors:** Ans Rensen, Margo M. van Mol, Ilse Menheere, Marjan D. Nijkamp, Ellen Verhoogt, Bea Maris, Willeke Manders, Lilian Vloet, Lisbeth Verharen

**Affiliations:** 1 Department of Emergency and Critical care, Faculty of Health, Behavior and Society, HAN University of Applied Sciences, Postbox 6960, 6503 GL Nijmegen, The Netherlands; 2 Department of Intensive Care, Erasmus MC, University Medical Center’s, Gravendijkwal 230, 3015 CE Rotterdam, The Netherlands; 3 Faculty of Psychology and Educational Sciences-Open University of the Netherlands, Valkenburgerweg 177, 6419 AT Heerlen, The Netherlands; 4 Medical Center Spaarne Gasthuis, Vondelweg 999, 2026 BW Haarlem, Netherlands; 5 Medical Center Gelderse Vallei, Willy Brandtlaan 10, 6716 RP Ede, The Netherlands; 6 IQ scientific Institute for Quality of Healthcare, Radboud University Medical Center, Nijmegen, The Netherlands

**Keywords:** Quality of healthcare, Consumer satisfaction, Intensive care unit, Relative, Family member, Consumer quality index

## Abstract

**Background:**

The quality standards of the Dutch Society of Intensive Care require monitoring of the satisfaction of patient’s relatives with respect to care. Currently, no suitable instrument is available in the Netherlands to measure this. This study describes the development and psychometric evaluation of the questionnaire-based Consumer Quality Index ‘Relatives in Intensive Care Unit’ (CQI ‘R-ICU’). The CQI ‘R-ICU’ measures the perceived quality of care from the perspective of patients’ relatives, and identifies aspects of care that need improvement.

**Methods:**

The CQI ‘R-ICU’ was developed using a mixed method design. Items were based on quality of care aspects from earlier studies and from focus group interviews with patients’ relatives. The time period for the data collection of the psychometric evaluation was from October 2011 until July 2012. Relatives of adult intensive care patients in one university hospital and five general hospitals in the Netherlands were approached to participate. Psychometric evaluation included item analysis, inter-item analysis, and factor analysis.

**Results:**

Twelve aspects were noted as being indicators of quality of care, and were subsequently selected for the questionnaire’s vocabulary. The response rate of patients’ relatives was 81% (*n* = 455). Quality of care was represented by two clusters, each showing a high reliability: ‘Communication’ (α = .80) and ‘Participation’ (α = .84). Relatives ranked the following aspects for quality of care as most important: no conflicting information, information from doctors and nurses is comprehensive, and health professionals take patients’ relatives seriously. The least important care aspects were: need for contact with peers, nuisance, and contact with a spiritual counsellor. Aspects that needed the most urgent improvement (highest quality improvement scores) were: information about how relatives can contribute to the care of the patient, information about the use of meal-facilities in the hospital, and involvement in decision-making on the medical treatment of the patient.

**Conclusions:**

The CQI ‘R-ICU’ evaluates quality of care from the perspective of relatives of intensive care patients and provides practical information for quality assurance and improvement programs. The development and psychometric evaluation of the CQI ‘R-ICU’ led to a draft questionnaire, sufficient to justify further research into the reliability, validity, and the discriminative power of the questionnaire.

**Electronic supplementary material:**

The online version of this article (doi:10.1186/s12913-016-1975-4) contains supplementary material, which is available to authorized users.

## Background

An Intensive Care Unit (ICU) is a place full of uncertainty and stress for both patients and for their relatives. Many researchers have suggested that relatives of ICU patients have a need for accessibility, support and information [1–3]. Nurses usually respond to these needs intuitively, based on individual experience, rather than in an evidence-based way [4]. Furthermore, relatives rarely ask for support and attention which may, in the long term, result in psychological distress [5, 6]. Therefore, it is necessary to better tailor the quality of care to relatives. Furthermore, according to the quality standards of the Dutch Society of Intensive Care (NVIC), every ICU needs to monitor the quality of care, including the satisfaction of the relatives with respect to the care [7, 8]. However, an evidence-based, valid, and reliable Dutch instrument that evaluates concrete experiences and perceived quality of care from the perspective of the relatives is still lacking.

To bridge the gap of this lacking instrument, we had some thoughtful considerations translating and adapting existing questionnaires. The ‘Critical Care Family Needs Inventory’ (CCFNI) is a questionnaire with 45 items to measure the needs of relatives in the ICU [9] in a French and an English version. However, *need* as a unique starting point is not sufficient to confirm simply and straightforward which interventions may have positive effects in the support of relatives [10]. Thus, the CCFNI does not adequately assess the quality of care as perceived by relatives. Another frequently used questionnaire to evaluate the satisfaction of the ICU patients’ relatives is the ‘Family Satisfaction in the ICU survey’ (FS-ICU) [11, 12]. Their items are based on an existing framework that measures patient satisfaction, in combination with items related to end-of-life care. The FS-ICU 24 seems a valid, reliable and feasible instrument for determining the *satisfaction* of relatives in ICU. Quite apart from the fact that satisfaction of patients might not at all correlate with the satisfaction of the relatives [13], it is preferable to measure *experiences* rather than satisfaction as they give more objective and specific information for quality improvement [14]. The utilized concept of satisfaction might raise some bottlenecks such as ceiling effects, cognitive dissonance and socially desirable answers. A discrepancy model, which describes satisfaction as a result of expectation minus the perceived experience, could overcome these problems [15]. Because of this conceptual difference, the FS-ICU was not used to translate and adapt the items of the questionnaire. The ‘Critical Care Family Satisfaction Survey’ (CCFSS) was assessed as a reliable and valid tool to measure the satisfaction of relatives as well [16]. Yet, both instruments, the FC-ICU 24 and the CCFSS, have a disadvantage when being implemented in the Netherlands, as they have been developed and used in a non-Dutch situation. Therefore, it is likely that some items will be rated as being more or less important by relatives in different countries or even on different continents [17]. For example, perceptions related to decision making might have fundamental culture specific differences on overall responsibilities of the medical team or the relatives. In addition, questions in this domain seemed multi-interpretable and difficult to translate in the exact meaning of the original questionnaire. Therefore, it was desirable to develop a measurement instrument that specifically evaluates the quality of care from the perspective of relatives in ICUs in the Netherlands in a logical follow-up of all previous studies.

This paper describes the development of a valid, reliable and feasible measuring instrument in the quality of care for practical use in ICUs in the Netherlands. The development process was based on standards for determining the experiences with provided care from a client group’s perspective, according to the Consumer Quality Index (CQI) method [18]. The CQI instruments are theoretically founded by the CAHPS® instruments and QUOTE® methodology, both based on a discrepancy model. To meet a sufficient quality of care, the expectations regarding the quality should be in accordance with the perceptions of the actual experiences according to these methodologies [15]. This questionnaire, the CQI ‘Relatives in Intensive Care Unit’ (CQI ‘R-ICU’), has been developed in a close cooperation between the University of Applied Sciences of Arnhem and Nijmegen, the Open University of the Netherlands and three hospitals (Erasmus University Medical Centre Rotterdam and the regional medical centers Kennemer Gasthuis Haarlem and Ziekenhuis Gelderse Vallei Ede). The Medical Ethics Committee of Erasmus MC judged that the research proposal (MEC-2011-189) complied with the Dutch law on Medical Research in Humans (WMO).

The strength of the CQI questionnaire is that it addresses the conceptual and methodological problems associated with satisfaction surveys, and that relatives were directly involved in the instrument’s development. The questionnaire focuses on “reports” of facts and experiences of the quality of care rather than on subjective ratings of satisfaction [14, 15, 19]. An important step in the development of a CQI is determining the measurable aspects of care (quality indicators), whereby many authors have adopted a structure, process and outcome indicator [20–22]. The aim of this study is to develop an appropriate set of quality indicators which measures all the domains in the quality of care relating to relatives in the ICU.

## Methods

### Questionnaire development of the CQI ‘R-ICU’

The overall research plan, based on the CQI Manual [18], consisted of qualitative and quantitative surveys. The method included four phases: 1) preparation, 2) performance, 3) psychometry, and 4) discriminatory. This CQI method has been described extensively in a previous study [15]. The current article, is limited to the first three phases. The Strobe guidelines have been used for preparing current article [23].

#### Preparation phase

The aim of the preparation phase was to detect relevant quality aspects of healthcare performance in the ICU in the support of relatives. A literature review was completed in Pubmed, Embase, Cinahl and Invert, including a search for existing questionnaires published in the period 2000–2011. Subsequently, five experts composed a topic list for focus group discussions with relatives of ICU-patients on their experiences in the ICU. Focus group discussions were conducted with a total of 18 Dutch speaking relatives of ICU patients aged 18 and older; in Erasmus MC (*n* = 7), Kennemer Gasthuis (*n* = 4), and Ziekenhuis Gelderse Vallei (*n* = 7). In each hospital, the same researcher (IM) acted as moderator during the focus group discussion. Each discussion was audiotaped; the transcriptions were coded and analyzed through thematic analysis [24] by four researchers.

#### The performance phase

Based on the results of the preparation phase, a questionnaire was drafted in the performance phase. Following the CQI Manual guidelines [18], the questionnaire consisted of two parts. Part one consisted of three types of questions covering experience, problems, and general judgment. The experience questions were either scored on a 4-point Likert scale (ranging from 1 = never, 2 = sometimes, 3 = often to 4 = always) or they were dichotomous (yes/no), as prescribed in the CQI Manual guidelines and forcing a non-neutral answer. The problem questions were scored on a 3-point scale (ranging from 1 = big problem, 2.5 = small problem, 4 = no problem), also according to the development guidelines and meant to gather insights of the size of the problem [18]. The general judgment questions were scored on a scale from 0 to 10 (0 = very bad, 10 = excellent). Demographic variables and questions regarding general health were added.

Part two determined the relative importance of the items in the questionnaire. Ordinary 5-point Likert scales tend to be highly skewed towards the ‘important’ dimension. Therefore, a greater differentiation on the positive side of the continuum was realized by a Likert scale using 4-point response choices: 1 = not important, 2 = of some importance, 3 = important, 4 = extremely important. This non-neutral solution was proven to be workable [25].

This first draft was sent to 11 relatives of ICU patients and to 21 ICU experts (doctors, nurses, social workers, spiritual counselors, psychologists) in the three hospitals where the cognitive test (pilot test) was held. The relatives should not experience any problems self-completing the questionnaire, and the items had to be relevant, unambiguous, understandable and useful. Based on their response, any unclear items in the CQI ‘R-ICU’ were rephrased.

#### Psychometric phase

In the third phase, the questionnaire was tested to assess the psychometric properties. In the period October 2011 - July 2012, the test was conducted in six hospitals: 4 ICUs in one university hospital, and 5 single ICUs in general hospitals. Inclusion criteria of the relatives were: age >18; Dutch language speakers; relative is partner or child/brother/sister, parent or other important relative of the ICU patient; patient stayed at the ICU ≥ 24 h; and the relative was present at admission at the ICU. Relatives were excluded when the patient died within 24 h after admission to the ICU. Most of the eligible relatives were approached to participate in the study. No data were available of relatives who were not met to include in the study, for example because the time of visiting was not matching to researcher’s working hours. After obtaining informed consent the questionnaire and cover letter were sent by post within 1 week of the patient’s discharge from the ICU. Up to two reminders were sent to non-responders after 1 and 4 weeks [26]. The recipients could return the questionnaire in a stamped, addressed envelope.

### Data analysis

The data were analyzed in order to identify item response rates and frequency distributions. Questionnaires completed by someone other than the relative, and questionnaire items with more than fifty percent of the answers missing, were excluded. Questionnaire items were excluded from further analysis if they had an item non-response of >5% of expected responses or an extreme skewness score (>90% of responses in the same category, i.e., a ceiling or floor effect). Spearman’s correlation coefficient was calculated to check for correlations between items (*r* > 0.70). Items with a negative wording were recoded to ensure comparability in the analysis. The answers on both the experience and the importance items were calculated in means and standard deviations to compare differences in the scores per item and to analyze significant differences between groups in the demographic variables.

#### Quality improvement scores

Quality improvement scores (QIS) were calculated by multiplying the importance scores with the percentages of the negative response categories ‘never’, ‘sometimes’, ‘big problem’ or ‘no’ on the corresponding experience questions. The improvement scores form an estimate of the potential improvement of quality of care and are useful for internal monitoring. Scores > 1.0 may potentially improve quality of care (range: 1–4).

#### Factor analysis

An exploratory factor analysis (EFA) was used to cluster the experience questions. In the first EFA, only the 27 experience items with a 4-point Likert scale were included. Preliminary requirements were Kaiser-Maier-Olkin Measure of Sampling Adequacy (KMO-value) ≥ 0.60 and the Barlett’s test of sphericity significant (*p* > .05), so that the number of respondents was sufficiently large and the correlations between the variables high enough to detect a factor loading. All factors with Eigenvalue ≥ 1.0 and factor loadings ≥ 0.30 were retained. The internal consistency of the different subscales was analyzed using Cronbach’s alfa, using α ≥ 0.70 as criterion for being reliable. In addition, we used the Item Total Correlation (ITC) ≥ 0.30 to define whether or not an item belonged to a certain subscale. All statistical analyses were performed using SPSS 18.0.

## Results

### Preparation and performance phase

The literature search in PubMed and EMbase resulted in 284 and 285 hits respectively; a search on PsycInfo, Invert and CINAHL did not lead to new aspects. Subsequently, adding ‘questionnaire’ in the title and abstract resulted in 43 articles. To explore relevant topics, we studied the Critical Care Family Needs Inventory (CCFNI) [9, 27], the Family Satisfaction in the Intensive Care Unit (FS-ICU 24/34) [12], the Critical Care Family Satisfaction Survey (CCFSS) [16], the Parent Satisfaction Instrument [28] and the CQI-palliative care relatives [29]. The resulting topic list with relevant quality aspects consisted of: support at first entrance in ICU, information and communication, attitude of the caregivers, (multidisciplinary) support, participation, organization of ICU, discharge to a general ward, and aftercare. Table 1 shows how the various aspects of the focus group discussion were divided among these quality indicators.

**Table 1:** Quality indicators and aspects: results from the qualitative phase

Quality indicator	Aspects
Structure	
Organization	Organization of the ICU
	▪ Patient room, waiting room, environment
	▪ Coordination between different disciplines
	▪ Possibilities to visit
	▪ Privacy
	▪ Noise
Process	
Communication	Informative communication
	▪ *Content*; treatment, prognosis, condition, situation, ICU
	▪ *Form*; oral, written, e-mail, digital
	▪ *Quality*; comprehensive, complete, open and honest, consistent, Listening attitude of caregiver
	Affective communication
	▪ Involvement
	▪ Attitude
	▪ Attention from caregivers
	▪ Take time for conversation and timely information
Care for relatives	Support at first entrance in ICU
	After care
	Psychosocial support
	▪ Emotional support
	▪ Spiritual/religious support
	▪ Practical support
Participation	Present during care or visit rounds
	Role for relatives in decision-making
	Being part of the care process
Outcome	
General judgement	Communication with nurses
	Communication with doctors
	Care and support in the ICU

**Fig. 1:**
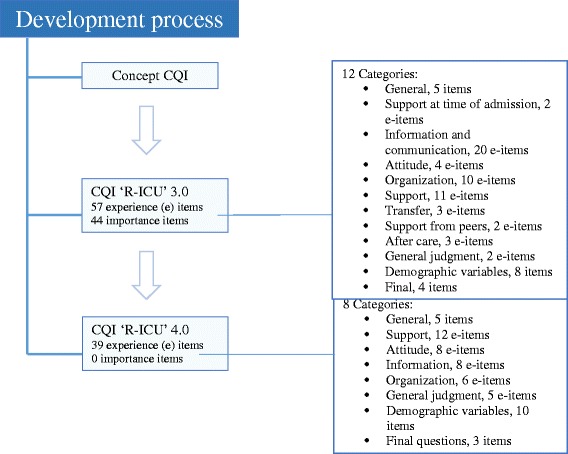
CQI ‘R-ICU’development process

The draft of the CQI ‘R-ICU’version 3.0, with the cognitive test feedback from relatives and the critical view of healthcare professionals, consisted of 74 questions divided into 12 categories: (1) general; (2) support at time of admission; (3) information and communication; (4) attitude; (5) ICU organization; (6) support; (7) transfer; (8) support from peers; (9) after care; (10) general judgment; (11) demographic variables, and (12) final questions. Of the total of 74 questions, 57 were constructed as so called ‘experience questions’. This resulted in a temporary set of 44 importance questions in part two of the CQI ‘R-ICU’(Fig. 1).

### Psychometric phase

In total, 564 relatives received the CQI ‘R-ICU’. Of these, 455 returned the questionnaire (response rate = 81%); four uncompleted questionnaires were excluded, as were five questionnaires which has been filled in by someone other than the relative. The dataset for the analysis contained 446 questionnaires. Relatives’ characteristics are presented in Table 2.

**Table 2:** Characteristics of the study sample (*n* = 446)

	Number	Percent
Relation to patient	441	
Partner	243	55.1%
Father/mother	46	10.4%
Son/daughter	97	22.0%
Brother/sister	35	7.9%
Other	20	4.6%
Gender	441	
Male	154	34.9%
Female	287	65.1%
Age	441	Modus 55–64
18–24	9	32.0%
25–34	23	5.2%
35–44	58	13.2%
45–54	107	24.3%
55–64	126	28.6%
65–74	91	20.6%
≥ 75	27	6.1%
Health indication	440	
Excellent/very good	177	40.2%
Good	223	50.7%
Moderate/poor	40	9.1%
Level of education	433	
No formal education (did not complete primary school)	9	2.1%
Primary education (primary school, special education in primary school)	21	4.8%
Lower secondary or preparatory vocational education (e.g., LTS, LEAO, LHNO, VMBO)	70	16.2%
Intermediate secondary vocational education (e.g., MAVO, [M]ULO, MBO-kort, VMBO-t)	87	20.1%
Senior secondary vocational education or work-based pathway (e.g., MBO-lang, MTS, MEAO, BOL, BBL, INAS)	84	19.4%
Senior general secondary education and university preparatory secondary education (e.g., HAVO, VWO, Atheneum, Gymnasium, HBS)	50	11.5%
Higher professional education (e.g., HBO, HTS, HEAO, HBO-V, academic education candidacy)	78	18.0%
Academic higher education (university)	29	6.7%
Other (please specify):	5	1.2%
Native country	441	
Dutch	407	92.3%
Other	34	7.7%
ICU-admission	442	
Planned	158	35.7%
Not planned	284	64.3%
Length of stay ICU (in days) (range 1–183)	432	Mean (SD) 13.2 (18.4)
Current situation	440	
Hospital	202	45.9%
Home	144	32.7%
Deceased	84	19.1%
Other	10	2.3%

#### Results of the experience questions on the ICU

Table 3 shows the results of the five highest and five lowest scores on the experience questions calculated in mean scores. The highest scores were calculated for the most positive category, and the lowest for the most negative category or the dichotomous response category. The mean score for the question General judgement for Care and Support in the ICU was 7.56 (SD = 1.83). A paired samples *t*-test showed a significant difference in the scores for Communication with nurses (M = 8.23, SD = 1.46) and Communication with doctors (M = 7.39, SD = 2.20) conditions; t(424) = −9.38, *p* = 0.000.

**Table 3:** Five of the highest and lowest mean scores in the experience questions

Five highest experience scores	Mean (SD)	Five lowest experience scores	Mean (SD)
Not affected by visit of relatives of other patients	3.85 (.40)	Hospital offers contact with peer group	1.07 (.45)
Not affected by noisiness of ICU-staff	3.78 (.52)	Information via digital means (email, website, electronic record)	1.11 (.49)
Not affected by changes in medical team	3.64 (.64)	Relatives can have contact with peers	1.24 (.81)
Professionals do not give conflicting information	3.59 (.71)	Information about the use of meal services in hospital	1.73 (1.12)
Information from nurses is understandable	3.56 (.66)	Information on writing in a diary	2.00 (1.30)

We used ANOVAs to test the differences between gender and age groups, relation to patient, length of stay at the ICU, planned or unplanned admission, deceased or not, and level of education, on the items ‘General judgment of the ICU’ and the two items ‘Communication with nurses and with doctors’. There was a significant difference between the different age groups on ‘General judgment of the ICU’ [F(2, 425) = 6.46, *p* = .002]. Ratings for the group ≥ 65 were higher (M = 8.02, SD = 1.6) than for the group 18–54 (M = 7.25, SD = 2.04). For the two overall items ‘Communication with doctors and nurses’, relatives rated a mean score of 7.39 (SD = 2.20) and 8.23 (SD = 1.46) respectively on a scale 0–10. We found a significant difference between the group survivors and non-survivors on the item ‘Communication with doctors’ [F(1, 419) = 5.13, *p* = .024]. The non-survivors group rated ‘Communication with doctors’ higher (M = 7.89, SD = 2.13) than the survivors group (M = 7.28, SD = 2.19). There was a significant effect on the item ‘Communication with nurses’ at the *p* < .01 level [F(2, 425) = 2.95, *p* = .053]. The ≥ 65 group rated ‘Communication with nurses’ higher (M = 8.41, SD = 1.43) than the 18–54 group (M = 8.03, SD = 1.55).

#### Results of the importance questions

In the first step of analysis of the importance study to select items for construction of the CQI ‘R-ICU’, the scores were calculated for questions with the response category ‘extremely important’ (score = 4) and the categories ‘not important/of some importance’ (score = ≤2). Respondents on the importance questions considered 12 items to be ‘extremely important’ and 8 items to be ‘not important/of some importance’. Table 4 represents the five most and the five least important items. In the next step, the quality improvement scores (QIS) were calculated by multiplying the mean of the importance scores (IS) with the percentages of the negative response categories ‘never/sometimes/big problem or no’ on the corresponding experience questions (EQ). The QIS gave an estimate for the potential improvement of quality of care and were useful for internal monitoring. Table 5 shows the top-10 of scores > 1.0.

**Table 4:** Five of the highest and lowest scores in the importance scores (%)

Five highest Importance scores (score = 4)	%	Five lowest Importance scores (score ≤ 2)	%
Do not give conflicting information	79	Hospital offers contact with peers	82
Information from doctors is understandable	72	Need for contact with peers	81
Information from nurses is understandable	70	Not affected by noise of equipment	70
Healthcare professionals treat relatives seriously	69	Contact with spiritual counselor	65
Nurses listen carefully to relatives	63	After care appointment with relative after discharge patient	55

**Table 5:** Top 10 of Quality Improvement Scores (QIS) >1.0

Quality aspect	IS^a^	IQ^a^	QIS^a^
Information how they can contribute to the care for the patient	3.18	2.06	2.21
Information via digital means (email, website, electronic record)	2.23	1.11	2.15
Information about the use of meal-facilities in the hospital	2.54	1.73	1.96
Involved in decisions on the medical treatment of patient	3.47	2.43	1.90
Information about parking facilities and any fees for this	2.79	2.01	1.88
The opportunity to contribute to patient care	3.05	2.34	1.80
The opportunity to be present during doctor’s visit to the patient	3.32	2.40	1.85
Written information during admission of the patient	2.62	2.01	1.81
The opportunity, after discharge of the patient, to talk with a professional about relatives experiences in the ICU	2.38	2.22	1.80
Have a fixed contact person to obtain information	3.40	2.46	1.74

^a^IS = importance scores; EQ = Experience questions; QIS = Quality Improvement Scores

### Factor analysis and internal consistency

According to the CQI guideline [18], 6 items were left out of the factor analyses. Five experience questions had a nonresponse >5%: accessibility fixed contact person; doctors listen carefully to relatives; family room fits needs; sufficient facilities to stay at ICU; and attention to mutual contact relatives. One item, ‘relatives get information through digital means’, was extremely skewed (>90%).

Factor analysis was carried out on 27 experience items with a 4 point Likert scale to determine the underlying structure of the newly developed questionnaire. The PCA met all the requirements, KMO excellent (0.88) and Bartlett’s test of sphericity significant (*N* = 364 en *p* < .000). The EFA (Table 6), based on 27 experience questions, showed a 2-factor-solution with an explained variance of 34.4%, covering all items (KMO 0.88, Bartlett’s test *p < 0.000*, *N* = 364). Two factors that best show the quality of care resulted from the EFA: Communication (α = .80) with 14 items, and Participation (α = .84) with 13 items.

**Table 6:** Domains, items and internal consistency of the second factor analysis (27 items)

		Factor loading	ITC	α if item deleted
Item No.	Factor 1 Communication (α = .80; *n* = 390)			
7	Prepared to first confrontation with patient	.45	.46	.79
9	Information given by doctors was understandable	.48	.51	.78
10	Information given by nurses was understandable	.61	.55	.78
11	Healthcare professionals did not give conflicting information	.42	.36	.80
28	Healthcare professionals take relatives seriously	.67	.59	.78
29	Healthcare workers have sufficient time	.69	.65	.77
30	Doctors listen carefully to relatives	.47	.52	.78
31	Nurses listen carefully to relatives	.62	.57	.78
32	Visiting hours connect to need relatives	.28	.29	.80
33	Not affected by changes in medical team	.48	.37	.79
34	Not affected by presence of sound of equipment	.51	.25	.80
36	Not affected by noisiness ICU-staff	.58	.36	.80
37	Not affected by visit of other patients	.28	.10	.81
38	Adequate opportunity for privacy on ICU	.41	.37	.80
Item No.	Factor 2 Participation (α = .84; *n* = 388)			
8	Written information during hospital stay	.35	.32	.84
14	Informed about professionals involved by healthcare	.45	.49	.83
16	Informed about working method on IC	.54	.59	.82
18	Information on writing in a diary	.44	.32	.84
20	Information on contributions to care	.64	.62	.82
21	Ability to contribute to care	.60	.53	.82
22	Opportunity to be present at doctor’s visit	.54	.51	.83
23	Involved in decision-making medical treatment	.50	.48	.83
24	Informed about parking and parking fees	.37	.28	.84
25	Informed about meal services	.50	.44	.831
35	Healthcare professionals explained why noise	.59	.52	.83
42	Attention to ‘how it is’ with relatives	.73	.69	.82
43	Attention to what relatives needed	.71	.69	.81

### CQI ‘R-ICU’ 4.0

Based on the results of the psychometric test, the CQI ‘R-ICU’, version 3.0 was revised following a pilot test and discussion by experts. Nine experience questions were reformulated: information about a fixed person; information which professionals contributed to care; presence of family waiting room in ICU; attention to relatives and their needs; coming in contact with other disciplines; information about transfer; and need for after care personal interview. The questions on the following 5 issues were deleted: nuisance of noises equipment; attention to contact between relatives; transfer a problem from ICU to other ward; information about support group; and need for contact with peers. In addition, the 5 ‘skip and go’ questions were deleted and the response category ‘inapplicable’ was added for the questions to which they belonged.

The CQI ‘R-ICU’ version 4.0 (see Additional file 1) consisted of 55 questions divided into 8 categories (Fig. 1): general (5 items); support (12 questions); attitude (8 items); information (8 items); organization (6 items); general judgment (5 items); demographic variables (10 items); and final questions (3 items). The CQI ‘R-ICU’ version 4.0 will be tested on a large scale in 20–30 ICUs in the discriminating phase of the study.

## Discussion

The aim of this study was the development and psychometric evaluation of a new Dutch questionnaire to measure experiences of ICU patients relatives’ with the quality of care, the CQI ‘R-ICU’. At first, the underlying aspects of the total care quality assessment for relatives of ICU patients were determined. From the focus group discussions it appeared that relatives found information on the patient’s situation of utmost importance. Furthermore, they considered the following aspects to be essential: to be involved, honest communication, way of approaching, attention, time of the health care providers, emotional support, participating in the process of caring, and being present during medical visit rounds. The participants ranked second in importance: care at first entrance, visiting hours, privacy, and waiting room.

The a-priori grouping, with the structure indicator ‘Organization’ and the process indicators ‘Communication’, ‘Care for relatives’ and ‘Participation’ were not found in the factor analyses. Alternatively, a clustering of items was found on two process factors; Communication and Participation. We need to note that these factors include the same items as preliminarily defined in the aspects of the quality indicators, although they also contain items of the structure indicator Organization (privacy, noise, waiting room, possibilities to visit). The items ‘noise’, ‘possibility to visit’, and ‘privacy’ showed a limited internal consistency (ICT < 0.3), and were consequently removed from the factor. The item ‘waiting room’ was excluded due to the number of missing values. The item ‘information about parking and parking fees’ was classified in the factor Participation, but it also had an ICT < 0.3. All these items seemed to fit with the factor Organization. When we performed a factor analysis with four factors, the factor Organization only included 2 items ‘privacy’ and ‘possibilities for visiting’ with a very low alpha (α = .49).

Following the CQI guideline, [18] several items (digital information, having fixed person, contact and support with other professionals, waiting room, transfer from ICU, support group, after care appointment) were not included in the factor analysis. These items were either not categorized as an experience question, skewed, had a high number of missing values because of ‘skip and go’ answers. Although some items (e.g., doctors listen carefully) had too many missing values, we had to include the item as they were required questions. Most of these items were reformulated and have been included in the CQI ‘R-ICU’ 4.0 for the discriminatory phase study, which will be more appropriate to determine its underlying structure.

The short length of stay of the patient at the ICU might have been another important reason for missing values. In these cases, relatives often indicated that they were not able to answer all the questions. In current study, relatives were included based on being admitted for ≥ 24 h at the ICU. In other studies, family of patients were included only when admitted for ≥48 h at the ICU [26] or >6 h [30]. This variable length of stay is important for the analysis of the discriminatory phase, in order to decide whether it is necessary to design a special CQI ’ R-ICU’ for the short-stay group.

In current study, the overall score of relatives’ experiences with the quality of care at the ICU was high, which matches findings from other studies [29–32]. Relatives were most satisfied about organizational and environmental aspects (e.g., no noise of staff, visiting hours, shift in medical staff, accessibility fixed person) and aspects of communication (e.g., no conflicting information, understandable) and they were dissatisfied about informational aspects (e.g., digital and written information, support groups, meals), participation aspects (e.g., care for patient, keeping diary) and supportive aspects (after care personal interview). Both in current and another study [29], relatives wanted the physician to be more available for regular person-to-person calls. It is noteworthy that the question ‘do doctors listen carefully’ had a high rate of missing values (6.1%); relatives noted that they had not seen the doctor. A possible explanation was that these patients were only admitted for 1 day and the relatives had missed the doctor’s visit. This item will be revised with a response category ‘did not see a doctor’.

The oldest group (>65) had a significantly higher overall score and a significantly higher score regarding the communication with nurses. The relatives of the non-survivors had a significantly higher score regarding the communication with doctors. These results correspond to results from other studies [33, 34], that recommend that younger patients and their families may need more support around end-of-life preparation and discussion of treatment preferences.

Current study showed that items related to contact with professionals for psychosocial care, aftercare and support groups, were relatively less important. In recent literature, the opposite was observed [35–37]. It may be possible that relatives only recognize the impact of the stressful period and the need for psychosocial care at a later stage [35]. Possible explanations for this may be the of measurement, the kind of relationship, severity of illness, and current situation of the patient.

### Practical implications

Two strengths of the development of the CQI ‘R-ICU’ questionnaire are that it attempts to overcome the conceptual and methodological problems associated with previous satisfaction surveys and that the relatives were directly involved in the instrument’s development. The questionnaire focuses on “reports” of the quality of care rather than on highly subjective ratings of satisfaction [15]. These individual-reported measures are essential to quality improvement programs as they will provide feedback regarding person-centeredness in daily practice to healthcare professionals and policy makers. It is therefore essential to involve the individual as an active partner in professional care and treatment [38]. This means seeing them as valuable persons, working alongside professionals to get the best outcome. The CQI ‘R-ICU’ is a helpful instrument to learn from the relatives as partners in caring for the ICU patient and use these reports to advance quality improvement effort in the ICU. Although this instrument has been developed in the Netherlands, the method could be applied in all healthcare settings in an international perspective. It seems a general and robust measurement instrument, even more applicable if the discriminative phase has been reported. This study, already conducted in 21 hospitals nationwide, will provide further knowledge on the applicability in different settings such as cardiology, general mixed, and specific ICUs. The translated English version of the CQI ‘R-ICU’ 5.0 can be found on http://blog.han.nl/acute-intensieve-zorg/files/2009/07/Engelse-vertaling-CQI-5.0-Naasten-op-de-IC-1.pdf.

### Limitations

Although the study had a strong design, with qualitative and quantitative result to rely on, some limitations to the results could be made. First, the respondents might have provided socially desirable answers, which were more positive than actually experienced. Second, a selection bias may have occurred, only the most satisfied individuals returned the questionnaire. Third, this study was performed in six hospitals resulting in a relatively small number of respondents to evaluate the whole population of ICU patients relatives’ in the Netherlands. Therefore, the results are not generally applicable. A nationwide multicenter study, which has already been conducted, is necessary to confirm or disapprove the identified results.

## Conclusion

The development and psychometric evaluation of the questionnaire-based Consumer Quality Index ‘Relatives in Intensive Care Unit’ (CQI ‘R-ICU’) resulted in a draft questionnaire that was sufficient to justify further research into the reliability, validity and the discriminative power of the questionnaire.

## Additional file

Additional file 1: Consumer Quality Index; Family Members in the Intensive Care Unit, version 4.0 March 2013. This is the questionnaire based on the results of the psychometric phase. (PDF 320 kb)

## Abbreviations

CCFNI: Critical care family needs inventory
CCFSS: Critical care family satisfaction survey
CQI: Consumer quality index
CQI ‘R-ICU’: Consumer Quality Index ‘Relatives in Intensive Care Unit’
EFA: Exploratory factor analysis
EQ: Experience questions
FS-ICU: Family satisfaction in the ICU survey
ICU: Intensive care unit
IS: Importance scores
ITC: Item total correlation
KMO-value: Kaiser-Maier-Olkin measure of sampling adequacy
NVIC: Dutch society of intensive care
QIS: Quality improvement scores
WMO: Dutch law on Medical Research in Humans
