# Supplementary material for: Quality of care in the intensive care unit from the perspective of patient’s relatives: development and psychometric evaluation of the consumer quality index ‘R-ICU’
Source: BMC Health Serv Res. 2017 Jan 24;17:77. doi: 10.1186/s12913-016-1975-4 (PMC5260020; doi:10.1186/s12913-016-1975-4)
Supplement: Additional file 1: — Consumer Quality Index; Family Members in the Intensive Care Unit, version 4.0 March 2013. This is the questionnaire based on the results of the psychometric phase. (PDF 320 kb) [file 12913_2016_1975_MOESM1_ESM.pdf]

Consumer Quality Index
Family Members in the Intensive Care Unit

Version 4.0
March 2013

Participant number ☐☐ ☐☐☐ ☐

This questionnaire was developed by the research department of Emergency and Critical Care, HAN University of Applied Sciences, in collaboration with Erasmus MC, Kennemer Gasthuis and the Gelderse Vallei Hospital.

The basic design of the CQI measuring instruments was developed by NIVEL, in collaboration with the Department of Social Medicine at the AMC.

This questionnaire is intended for family members (partners, relatives, close friends) of patients who have been admitted to the Intensive Care Unit (ICU). We will use your answers to identify the aspects in which we can improve our interaction with and counselling of family members in the ICU.

We would appreciate it greatly if you would complete this questionnaire. Participation in this study is voluntary. It will take around 15 minutes to complete the questionnaire. We ask that you complete it based on <u>your own</u> experiences, and that you do not consult with anyone else.

All information will be treated with the strictest confidence. Your personal information will not be shared with anyone. The health care professionals and insurance companies will not have access to your answers.

Additional information can be obtained from the hospital's contact person. Contact information is provided on the last page of this questionnaire.

If you choose not to complete this questionnaire, please place an X in this box ☐ and return this page in the response envelope (no postage is required).

INSTRUCTIONS FOR COMPLETING THIS QUESTIONNAIRE

- This questionnaire concerns the hospital's interactions with and counselling of family members of ICU patients.
- It concerns your perceptions of your interactions with professionals in the ICU and the counselling you received as a family member. You should therefore not complete it based on what the patient or other family members think. It concerns what you saw, heard or experienced yourself.
- Please answer all questions by placing an X in the box to the left of your answer.
- In some cases, you will be asked to skip a question.

  ☐ no

  ☐ yes → ***Please proceed to Question….***
- If you would like to change an answer, place brackets around the box you checked and choose another answer, as shown below:

  (X) No (wrong answer)

  X Yes
- It is important for the study that you complete the questionnaire as fully as possible, without skipping any questions.

INTRODUCTION

We begin by asking a few general questions about the patient's admission to the ICU.

1. Has one of your family members been admitted to an ICU within the past three months?

- ❑ No → ***This questionnaire does not apply to you. We kindly request that you return the questionnaire in the enclosed envelope. (No postage is required.)***
- ❑ *Yes*

2. In which hospital was the patient (your family member) admitted to the ICU?
If the patient was admitted to more than one ICU, please select the one where the patient stayed for the longest time.

……………………………………………..

(Please use block letters.)

3. How many days did the patient stay in the ICU?

……………………………… days.

4. Did the admission to the ICU come as a surprise to you?

- ❑ Yes
- ❑ No

5. What is the current situation?

- ❑ The patient has been admitted (to this hospital or another care institution).
- ❑ The patient is at home.
- ❑ The patient has died.
- ❑ Other (please specify):

(Please use block letters.)

The following questions concern your experiences related to your interactions with professionals in the ICU and the counselling you received in the ICU in the past 3 months. If the patient was admitted to more than one ICU, please refer to the ICU that you indicated in Question 2 when answering these questions.

In these questions, the term *professionals* refers to all professionals working in the ICU. Some questions refer to one specific professional (e.g. the nurse). If this is the case, it is indicated in the question.

COUNSELLING

The following questions concern the counselling that you received in the ICU as a family member in the past 3 months.

6. Was a professional available to help you during your first visit to the patient in the ICU?

- ❑ No
- ❑ Yes

7. Did the professionals prepare you for your first encounter with the patient during the admission?

- ❑ No, not at all
- ❑ Somewhat
- ❑ Largely
- ❑ Yes, completely

8. Did you receive information about how you could contribute to the patient's care?

- ❑ Never
- ❑ Occasionally
- ❑ Usually
- ❑ Always

9. Did the nurses give you the opportunity to contribute to the patient's care?

- ❑ Never
- ❑ Occasionally
- ❑ Usually
- ❑ Always

10. Did you have the opportunity to be present when the doctor visited the patient?

- ❑ Never
- ❑ Occasionally
- ❑ Usually
- ❑ Always

11. Did you feel as if you had a voice in decision-making about the patient's medical treatment?

- ❑ Never
- ❑ Occasionally
- ❑ Usually
- ❑ Always

12. Did the professionals explain the reasons for the sounds coming from the equipment?

- ❑ No, not at all
- ❑ Somewhat
- ❑ Largely
- ❑ Yes, completely

13. Did anyone pay attention to <u>your</u> needs?

- ❑ Never
- ❑ Occasionally
- ❑ Usually
- ❑ Always

14. Did you perceive your contact with the social worker as supportive?

- ❑ No, not at all
- ❑ Somewhat
- ❑ Largely
- ❑ Yes, completely
- ❑ Not applicable: I did not have contact with a social worker.

15. Did you perceive your contact with the chaplain as supportive?

- ❑ No, not at all
- ❑ Somewhat
- ❑ Largely
- ❑ Yes, completely
- ❑ Not applicable: I did not have contact with a chaplain.

16. Did you perceive your contact with the psychologist as supportive?

- ❑ No, not at all
- ❑ Somewhat
- ❑ Largely
- ❑ Yes, completely
- ❑ Not applicable: I did not have contact with a psychologist.

17. After the patient was discharged or died, did you have the opportunity to talk with a professional about your experiences in the ICU?

- ❑ No, not at all
- ❑ Somewhat
- ❑ Largely
- ❑ Yes, completely

TREATMENT

The following questions concern the <u>contacts</u> that you had with the <u>professionals</u> in the ICU in the past 3 months. The term *professionals* refers to all professionals working in the ICU.

18. Did <u>doctors</u> give you information in a manner you could understand?

- ❑ Never
- ❑ Occasionally
- ❑ Usually
- ❑ Always

19. Did <u>nurses</u> give you information in a manner you could understand?

- ❑ Never
- ❑ Occasionally
- ❑ Usually
- ❑ Always

20. Did professionals give you contradictory information?

- ❑ Never
- ❑ Occasionally
- ❑ Usually
- ❑ Always

21. Did professionals take you seriously?

- ❑ Never
- ❑ Occasionally
- ❑ Usually
- ❑ Always

22. Did professionals have enough time for you?

- ❑ Never
- ❑ Occasionally
- ❑ Usually
- ❑ Always

23. Did <u>doctors</u> listen carefully to you?

- ❑ Never
- ❑ Occasionally
- ❑ Usually
- ❑ Always
- ❑ Not applicable: I did not speak to a doctor.

24. Did <u>nurses</u> listen carefully to you?

- ❑ Never
- ❑ Occasionally
- ❑ Usually
- ❑ Always

25. Did you perceive disruption due to shift changes in the medical team?

- ❑ Never
- ❑ Occasionally
- ❑ Usually
- ❑ Always

INFORMATION

The following questions address various aspects of <u>information</u> concerning the patient's admission to the ICU. They refer to information concerning procedures in the ICU and the patient's health situation.
The questions concern the admission of your family member to the ICU in the past 3 months.

26. Did you receive a brochure containing general information about the ICU (e.g. telephone numbers, visiting hours and procedures)?

- ❑ No
- ❑ Yes

27. During the patient's admission, did you receive information digitally (e.g. email, website, electronic patient file)?

- ❑ No
- ❑ Yes

28. Did you know the roles of the professionals involved?

- ❑ Never
- ❑ Occasionally
- ❑ Usually
- ❑ Always

29. Did professionals inform you about maintaining a journal during the ICU period?

- ❑ No
- ❑ Yes

30. Did professionals inform you about social work, spiritual care or psychological assistance for yourself? *(you may provide more than one answer)*

- ❑ No
- ❑ Yes, about social work
- ❑ Yes, about spiritual care
- ❑ Yes, about psychological assistance

31. Were you informed about parking facilities and parking fees (if any)?

- ❑ No
- ❑ Yes

32. Were you informed about the use of food service facilities in the hospital (e.g. sandwiches, hot meals)?

- ❑ No
- ❑ Yes

33. Were you well informed in advance about the patient's transfer to another department or hospital/care institution?

- ❑ No, not at all
- ❑ Somewhat
- ❑ Largely
- ❑ Yes, completely

ORGANISATION

The following questions concern the organisation and surroundings of the ICU as you perceived them in the past 3 months.

34. Was a permanent contact person available to you?

- ❑ Never
- ❑ Occasionally
- ❑ Usually
- ❑ Always
- ❑ Not applicable: I did not have a permanent contact person.

35. Which other professionals did you have problems contacting? *(you may provide more than one answer)*

- ❑ Social workers
- ❑ Chaplains
- ❑ Psychologists
- ❑ Not applicable: I did not try to contact other professionals.

36. Did you have sufficient opportunity for privacy with the patient in the ICU?

- ❑ Never
- ❑ Occasionally
- ❑ Usually
- ❑ Always

37. Were you disturbed by other patients' visitors in the ICU?

- ❑ Never
- ❑ Occasionally
- ❑ Usually
- ❑ Always

38. Did the family room meet your needs?

- ❑ No, not at all
- ❑ Somewhat
- ❑ Largely
- ❑ Yes, completely
- ❑ Not applicable: There was no family room/I did not use the family room.

39. Did the visiting hours correspond to your needs?

- ❑ No, not at all
- ❑ Somewhat
- ❑ Largely
- ❑ Yes, completely

GENERAL ASSESSMENT

40. On a scale from 0 to 10, how would you rate the doctors in the ICU with regard to their way of communicating?

- ❑ 0 Extremely poor
- ❑ 1
- ❑ 2
- ❑ 3
- ❑ 4
- ❑ 5
- ❑ 6
- ❑ 7
- ❑ 8
- ❑ 9
- ❑ 10 Excellent

41. On a scale from 0 to 10, how would you rate the nurses in the ICU with regard to their way of communicating?

- ❑ 0 Extremely poor
- ❑ 1
- ❑ 2
- ❑ 3
- ❑ 4
- ❑ 5
- ❑ 6
- ❑ 7
- ❑ 8
- ❑ 9
- ❑ 10 Excellent

42. If you could change one thing about your interactions with the professionals in the ICU and the counselling you received, what would it be?

(Please use block letters.)

43. How would you rate the interactions with and counselling of family members in the ICU?

- ❑ 0 Extremely poor
- ❑ 1
- ❑ 2
- ❑ 3
- ❑ 4
- ❑ 5
- ❑ 6
- ❑ 7
- ❑ 8
- ❑ 9
- ❑ 10 Excellent

44. Would you recommend this ICU to other family members who must be admitted to an ICU?

- ❑ Absolutely not
- ❑ Probably not
- ❑ Probably
- ❑ Absolutely

PERSONAL INFORMATION

The following questions concern your personal situation. We are asking these questions to gain insight into the experiences of family members in different groups. This refers to differences in aspects such as age, education and ethnicity. Your answers will help us improve the coordination of interaction with and counselling of family members in line with such differences.

45. What is your relationship to the ICU patient?

I am his/her:

- ❑ Partner/spouse
- ❑ Father/mother
- ❑ Son/daughter
- ❑ Brother/sister
- ❑ Other (please specify):

(Please use block letters.)

46. Are you male or female?

- ❑ Male
- ❑ Female

47. What is your age (please write a number)?

……………………………………………

48. How would you describe your health in general?

- ❑ Excellent
- ❑ Very good
- ❑ Good
- ❑ Moderate
- ❑ Poor

49. What is the highest level of formal education you completed?
(completed means you earned a diploma or a certificate of satisfactory completion)

- ❑ No formal education (did not complete primary school)
- ❑ Primary education (primary school, special education in primary school)
- ❑ Secondary education (secondary school, high school)
- ❑ Post-secondary vocational education (trade college, technical school)
- ❑ Academic higher education (university)
- ❑ Other (please specify):

(Please use block letters.)

50. In which country were you born?

- ❑ Netherlands
- ❑ Indonesia/former Dutch East Indies
- ❑ Suriname
- ❑ Morocco
- ❑ Turkey
- ❑ Germany
- ❑ (former) Netherlands Antilles
- ❑ Aruba
- ❑ Other (please specify):

(Please use block letters.)

51. In which country was your mother born?

- ❑ Netherlands
- ❑ Indonesia/former Dutch East Indies
- ❑ Suriname
- ❑ Morocco
- ❑ Turkey
- ❑ Germany
- ❑ (former) Netherlands Antilles
- ❑ Aruba
- ❑ Other (please specify):

(Please use block letters.)

52. In which country was your father born?

- ❑ Netherlands
- ❑ Indonesia/former Dutch East Indies
- ❑ Suriname
- ❑ Morocco
- ❑ Turkey
- ❑ Germany
- ❑ (former) Netherlands Antilles
- ❑ Aruba
- ❑ Other (please specify):

(Please use block letters.)

53. Did anyone assist you in completing this questionnaire?

- ❑ No → ***go to question 55***
- ❑ *Yes*

54. How did this individual assist you? You may select more than one answer.

- ❑ Read the questions to me
- ❑ Wrote my answers
- ❑ Answered the questions on my behalf
- ❑ Translated the questions into my language
- ❑ Assisted in another way (please specify):

(Please use block letters.)

55. We aim to continue improving the questionnaire, so we would like to hear your opinion of it. Was anything missing from this questionnaire? Do you have any comments or suggestions? You may write them in the space below.

(Please use block letters.)

Many thanks for completing this questionnaire!

You can use the enclosed reply envelope to return the completed questionnaire.

If you would like to talk with someone about your experiences in the ICU as a result of this questionnaire, please contact:…
